# The Laser Welding Research of Dissimilar Materials Between AlCoCrFeNi2.1 Eutectic High-Entropy Alloy and GH3030 Nickel-Based Alloy

**DOI:** 10.3390/ma18214970

**Published:** 2025-10-31

**Authors:** Anmin Liu, Ze An, Bin Wang, Hailin Qiao, Keming Chang, Yu Fan

**Affiliations:** 1 School of Intelligent Equipment, Changzhou College of Information Technology, Changzhou 213164, China; 2 School of Materials Science and Physics, China University of Mining and Technology, Xuzhou 221116, China; 3 Nanjing Zhongke Shenguang Technology Co., Ltd., Nanjing 210028, China; 4 Jiangsu Weite Gaoke Welding Co., Ltd., Huai’an 223100, China

**Keywords:** laser material processing, fiber laser, high-entropy alloys, welding microstructure

## Abstract

Dissimilar material welding enables the integration of the superior properties of different materials, thereby achieving optimal structural performance and economic efficiency while meeting specific service requirements. The presence of solid-solution strengthening elements such as Ti, Co, and Al, and trace elements such as P and S, in GH3030 nickel-based superalloy leads to their segregation and the formation of intermetallic compounds in the welded joint, resulting in deterioration of joint performance. High-entropy alloys (HEAs), with their high-entropy effect and delayed diffusion effect working synergistically, can effectively suppress compositional segregation caused by uneven elemental diffusion and the formation of intermetallic compounds at interfaces, thereby improving the quality of welded joints and demonstrating great potential for dissimilar material joining. Therefore, in this study, fiber laser welding was used to effectively join AlCoCrFeNi2.1 eutectic high-entropy alloy and GH3030 nickel-based superalloy, with the expectation to improve welded joint element segregation, suppressing the formation of intermetallic compounds, and enhance the welded joint quality and its performance. The AlCoCrFeNi2.1/GH3030 joint exhibits an average yield strength of 1.31 GPa, which is significantly higher than that of the GH3030/GH3030 joint (1.07 GPa). In addition, the AlCoCrFeNi2.1/GH3030 joint shows a higher average work-hardening exponent of 0.337 compared with 0.30 for the GH3030/GH3030 joint, indicating improved plasticity. The results showed that under appropriate welding process parameters, the hardness of the weld zone, transitioning from the nickel-based superalloy to the eutectic high-entropy alloy, exhibited a stable increasing trend, and the joint exhibits good plasticity, with brittle fracture being unlikely.

## 1. Introduction

With the continuous development of science and technology, high-entropy alloys (HEAs) have become a popular research topic in the field of materials science due to their unique microstructures and excellent mechanical properties [1,2,3]. High-entropy alloys generally exhibit body-centered cubic (BCC), face-centered cubic (FCC), hexagonal close-packed (HCP), or multiphase solid-solution structures [4,5]. Based on their solid solution structures, HEAs exhibit outstanding properties such as excellent corrosion resistance, wear resistance, and strength. Additionally, they are less prone to defect formation under high radiation doses, making them promising materials for applications in nuclear energy, efficient thermal power generation, and dissimilar metal welding. Single-phase FCC HEAs have good ductility but low strength, while single-phase BCC HEAs exhibit high strength but poor ductility [6,7]. Balancing strength and plasticity in single-phase solid solution HEAs is challenging, which limits their industrial applications. However, studies have shown that eutectic structures offer good mechanical properties [8]. Lu et al. [9] first introduced the concept of eutectic high-entropy alloys (EHEAs) and found that the AlCoCrFeNi2.1 HEA, which has an eutectic composition and is composed of FCC and B2 phases with different hardness levels, can achieve a good balance of strength and plasticity. Research has shown that the AlCoCrFeNi2.1 eutectic high-entropy alloy has higher ultimate tensile strength and better elongation and demonstrates higher tensile strength at elevated temperatures [10]. Therefore, AlCoCrFeNi2.1 eutectic high-entropy alloy holds great potential for high-temperature applications.

Nickel-based alloys exhibit excellent thermal stability [11,12,13], and GH3030 nickel-based superalloy (80Ni-20Cr), as a solid solution-strengthened high-temperature alloy, demonstrates satisfactory thermal strength and high plasticity below 800 °C. It is a key material for manufacturing components such as turbine blades and engine combustion chambers, with numerous applications in aerospace and automotive industries [14]. However, research has shown that, on one hand, nickel-based superalloys contain a variety of solid-solution strengthening elements, such as W, Mo, Cr, Co, Al, Ti, etc., along with trace elements like C, B, Mg, P, S, and rare earth elements. These elements make the alloys prone to defects such as weld metal segregation, intermetallic compound reaction layers, and welding hot cracks, which lead to the fracture of the welded joints [15]. On the other hand, after laser welding, the high cooling rate may lead to the formation of some micropores and cracks [16]. This limits the development of GH3030 nickel-based superalloy as a high-temperature load-bearing component in laser welding applications.

The high-entropy effect of high-entropy alloys (HEAs) can suppress the formation of intermetallic compounds. Specifically, HEAs have higher configurational entropy than traditional alloys, which facilitates the formation of simple, disordered solid solutions rather than complex intermetallic compounds during solidification [17]. The sluggish diffusion effect in HEAs, due to the complexity of their chemical composition and the severity of lattice distortion, makes atomic diffusion within the alloy difficult, which helps prevent element segregation and the formation of intermetallic compound reaction layers at the interface during dissimilar metal welding [18]. Sonar et al. [19] investigated the effects of an EHEA interlayer on the microstructure and mechanical properties of dissimilar P91/304L steel joints. The results revealed that the introduction of the EHEAs interlayer imparted a high-entropy effect to the weld, leading to increased mixing entropy and retarded diffusion behavior. Consequently, the weld metal exhibited improved compositional homogeneity and a functionally graded structure, which minimized the unmixed zone, suppressed carbon migration, and reduced the peak hardness at the weld interface. Afonso et al. [20] successfully performed dissimilar laser welding between rolled CoCrFeMnNi high-entropy alloy (HEA) and Inconel 718 nickel-based superalloy, achieving a defect-free joint with a tensile strength of 822 MPa and an elongation at fracture of 7.1%. The synergistic effects of the high-entropy effect and sluggish diffusion effect give HEAs great potential for dissimilar metal welding, as they can suppress the formation of brittle intermetallic compounds at the interface, thereby improving weld joint quality. Zhang et al. reviewed the studies on the microstructure and properties of laser-melted HEAs and found that the characteristics of rapid laser melting and fast cooling can enhance the microstructure of HEAs [21]. Related studies have shown that laser oscillation and the composition of the protective gas can effectively improve the weld metal microstructure [22]. In addition, nanoindentation is an advanced technique for analyzing the mechanical properties of dissimilar joints. Mahto et al. [23] utilized nanoindentation to determine the elastic strain and strain-hardening coefficient of dissimilar joints between AA6061-T6 aluminum alloy sheets and AISI 304 stainless steel. Therefore, this study aims to address the issues of interface segregation and intermetallic compound formation during the welding of GH3030 by leveraging the synergistic effects of the high-entropy effect and sluggish diffusion effect in HEAs, optimizing weld joint performance through dissimilar metal welding.

## 2. Materials and Methods

### 2.1. Preparation of High-Entropy Alloys and Nickel-Based Superalloys

In this experiment, high-purity metallic powders with a purity greater than 99.95 wt% were used as raw materials. The elements Al, Co, Cr, Fe, and Ni were weighed in a molar ratio of 1:1:1:1:2.1, with a total mass of approximately 100 g. First, the raw materials were placed in a copper crucible according to the formulation. The vacuum electric arc melting furnace was then sealed, and the vacuum was applied. Subsequently, high-purity argon gas (Ar) was introduced into the furnace. The melting arc gun was adjusted to be positioned directly above the raw materials, and the power supply was turned on to initiate arc melting, ensuring complete melting of the materials. After a period of melting, the power supply was turned off, and the materials were allowed to cool naturally. The raw materials were flipped over, and melting continued to ensure uniform composition in the high-entropy alloy ingot.

Table 1 shows the composition of the high-entropy alloy. Based on the total mass of the high-entropy alloy, the amount of each metal required was calculated. Before weighing, the raw materials were cleaned and dried using an ultrasonic cleaner. The materials were then weighed using an electronic balance with a precision of 0.1 mg.

Table 2 shows the chemical composition of the nickel-based high-temperature alloy. Prior to the experiment, the GH3030 nickel-based alloy was cut into thin plates with dimensions of 10 mm × 10 mm × 2 mm using a wire cutting machine. To reduce welding defects, an acetone solution was used to remove oil contamination, followed by water washing and drying.

### 2.2. Laser Welding Process and Method

Before welding, the melted ingots of AlCoCrFeNi2.1 eutectic high-entropy alloy and GH3030 nickel-based high-temperature alloy were cut into thin plates using a wire cutting machine. The surfaces were polished with sandpaper to remove oil contamination and impurities. For the single-pass butt welding of AlCoCrFeNi2.1 eutectic high-entropy alloy and GH3030 nickel-based high-temperature alloy, no filler wire was used. To ensure the flatness and prevent deformation after welding, fixtures were used to fix the materials in place. Argon gas with a pressure of 0.15 MPa was used as the protective gas during the welding process.

**Figure 1:**
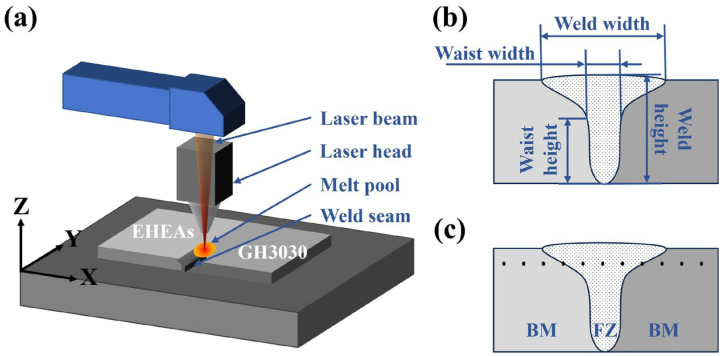
(**a**) Schematic diagram of the dissimilar laser welding process; (**b**) Schematic diagram of the welded joint morphology parameters; (**c**) Schematic diagram of hardness testing (BM–Base material, FZ–Fusion zone).

Based on the research on the influence of laser welding parameters on the welded joint of AlCoCrFeNi2.1 eutectic high-entropy alloy and GH3030 nickel-based high-temperature alloy, the study investigates the laser welding characteristics of dissimilar alloys between AlCoCrFeNi2.1 eutectic high-entropy alloy and GH3030 nickel-based high-temperature alloy. In this work, laser welding was conducted on GH3030/GH3030 nickel-based superalloy, AlCoCrFeNi2.1/AlCoCrFeNi2.1 eutectic high-entropy alloy, and AlCoCrFeNi2.1/GH3030 dissimilar materials. The AlCoCrFeNi2.1/GH3030 dissimilar-materials laser-welding process is illustrated in Figure 1a. In addition, the effect of different heat inputs (HI) on the AlCoCrFeNi2.1/GH3030 dissimilar joint was investigated. Since the heat input is directly related to laser power, welding speed, and welding efficiency, it can be calculated as HI = P/v, where P is the laser power and v is the welding speed. The welding parameters used in this study are listed in Table 3.

### 2.3. Welded Joint Morphology and Microstructure Analysis

A macroscopic inspection was conducted on the welded joint surface to observe the appearance of the weld formation, checking for any macro defects such as pores, cracks, or incomplete fusion. A camera was used to photograph the welded joint, and the weld surface formation patterns under different welding parameters for each material were analyzed.

The welded samples were cut into small pieces (10 mm × 10 mm × 2 mm) along a direction perpendicular to the welded joint using a wire-cutting machine. The samples were then embedded using a thermal embedding method. The weld joints were polished sequentially with sandpaper, from #80 to #2000 grit, and then placed on a polishing machine to achieve a mirror-like finish without scratches. The polished surfaces were etched with aqua regia until the gloss was lost. Afterward, the samples were cleaned with water and alcohol and dried by blowing air. The microstructure of the welded samples was observed using an Olympus-GX53 optical microscope (Olympus Corporation, Hachioji, Tokyo, Japan), and the welded joint morphology parameters were measured at 50× magnification. The specific welded joint morphology parameters are shown in Figure 1b. For high magnification microstructural observation, a Quanta 250 scanning electron microscope (SEM, Thermo Fisher Scientific, Waltham, MA, USA) was used. SEM-EDS line scans and area scans were performed to analyze the distribution of elements and changes in composition.

### 2.4. Phase Analysis

X-ray diffraction (XRD, Bruker AXS GmbH, Karlsruhe, Germany) was employed to detect the phase composition of the welded joint and base material. Prior to testing, the sample surface was cleaned to avoid contamination from surface impurities. X-ray diffraction was performed using Cu Kα characteristic radiation (average wavelength of 1.5418 Å) over a 2θ range of 20–80°. Phase identification was carried out using Jade 6 software by comparing the obtained diffraction patterns with standard powder diffraction data (PDF) provided by the Joint Committee on Powder Diffraction Standards–International Centre for Diffraction Data (JCPDS–ICDD).

### 2.5. Microhardness Testing

Microhardness testing was carried out on the Base material, heat-affected zone (HAZ), and fusion zone (FZ) using a Wilson VH1102 semi-automatic hardness tester (Buehler, Lake Bluff, IL, USA). The testing intervals were 0.5 mm, with a loading time of 15 s and a load of 200 gf. The locations for hardness measurement are shown in Figure 1c.

### 2.6. Nanoindentation Testing

Nanoindentation testing was performed on the welded joint and base material using an Anton Paar NHT2 nanoindentation instrument (Anton Paar GmbH, Graz, Austria), equipped with a Berkovich diamond indenter. Prior to testing, the sample surface was polished to ensure that any large scratches did not affect the results. During the test, a loading speed of 20.00 mN/min was set, and the indenter was pressed into the sample until a maximum load of 10.00 mN was reached. The load was held at the maximum for 10 s. During this period, hardness, elastic modulus, and creep strain index were calculated.

## 3. Results and Discussion

### 3.1. Effect of Process Parameters on Welded Joint Morphology of Eutectic High-Entropy Alloy and GH3030 Nickel-Based High-Temperature Alloy

**Figure 2:**
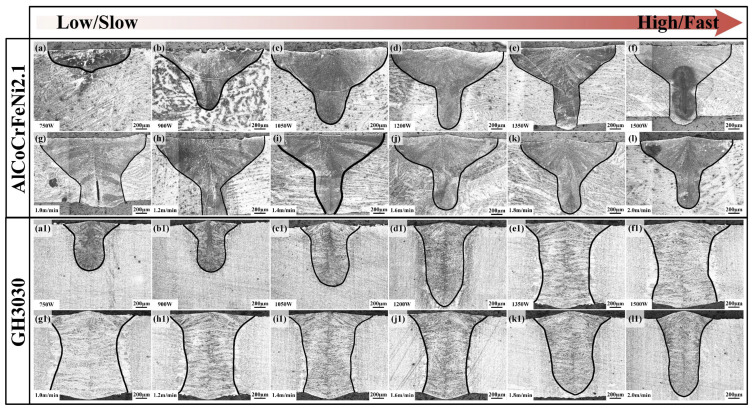
Welded Joint Morphology Images: (**a**–**f**) Welded joint morphology of AlCoCrFeNi2.1 eutectic high-entropy alloy under different laser power settings; (**g**–**l**) Welded joint morphology of AlCoCrFeNi2.1 eutectic high-entropy alloy under different welding speeds; (**a1**–**f1**) Welded joint morphology of GH3030 nickel-based high-temperature alloy under different laser power settings; (**g1**–**l1**) Welded joint morphology of GH3030 nickel-based high-temperature alloy under different welding speeds.

Figure 2 shows the welded joint morphology of AlCoCrFeNi2.1 eutectic high-entropy alloy and GH3030 high-temperature nickel-based alloy under different laser power and welding speed settings. It is evident that, compared to GH3030, AlCoCrFeNi2.1 eutectic high-entropy alloy exhibits a wider weld width and a narrower neck width under the same welding parameters. The AlCoCrFeNi2.1 eutectic high-entropy alloy exhibited a distinct “nail-shaped” weld morphology, characterized by a wider upper weld region and a narrower, deeper lower section with a relatively flat top surface. In contrast, the GH3030 superalloy showed more moderate variations in weld width and penetration depth, primarily due to the differences in thermal conductivity between the two materials. According to relevant reports, the thermal conductivity of the AlCoCrFeNi2.1 eutectic high-entropy alloy is approximately 17.5 W/(m·K) [24,25], while that of GH3030 nickel-based high-temperature alloy is 25.1 W/(m·K) [26]. The higher the thermal conductivity, the faster the heat transfer, which means that materials with lower thermal conductivity are more likely to form “nail-like” welded joints during laser welding [27].

**Figure 3:**
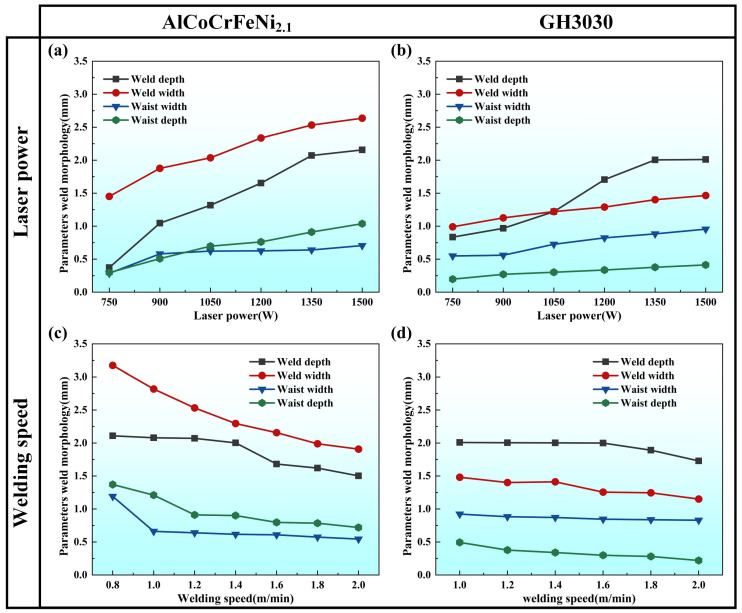
(**a**) Welded joint morphology parameters of AlCoCrFeNi2.1 eutectic high-entropy alloy under different laser power settings; (**b**) Welded joint morphology parameters of GH3030 nickel-based high-temperature alloy under different laser power settings; (**c**) Welded joint morphology parameters of AlCoCrFeNi2.1 eutectic high-entropy alloy under different welding speeds; (**d**) Welded joint morphology parameters of GH3030 nickel-based high-temperature alloy under different welding speeds.

Figure 3 shows a comparison of the welded joint morphology parameters of AlCoCrFeNi2.1 eutectic high-entropy alloy and GH3030 high-temperature nickel-based alloy under different laser power and welding speed settings. Figure 3a,b show the welded joint morphology parameters of AlCoCrFeNi2.1 eutectic high-entropy alloy and GH3030 high-temperature nickel-based alloy under different laser powers. The trend of laser power’s effect on the neck depth and neck width for both materials is relatively steady. However, as the laser power increases, both the weld depth and weld width of the eutectic high-entropy alloy show a significant upward trend, while the increase in weld width for GH3030 is more gradual. Figure 3c,d show the welded joint morphology parameters of AlCoCrFeNi2.1 eutectic high-entropy alloy and GH3030 high-temperature nickel-based alloy under different welding speeds. Both materials exhibited a similar decreasing trend in morphological parameters with increasing welding speed. However, when the welding speed increased from 1 m/min to 2 m/min, the weld width of the AlCoCrFeNi2.1 eutectic high-entropy alloy decreased by 0.91 mm, whereas that of GH3030 decreased by only 0.33 mm. Therefore, the variation in weld width was more pronounced in the AlCoCrFeNi2.1 eutectic high-entropy alloy than in GH3030.

### 3.2. Microstructural Analysis of AlCoCrFeNi2.1/GH3030 Joints

**Figure 4:**
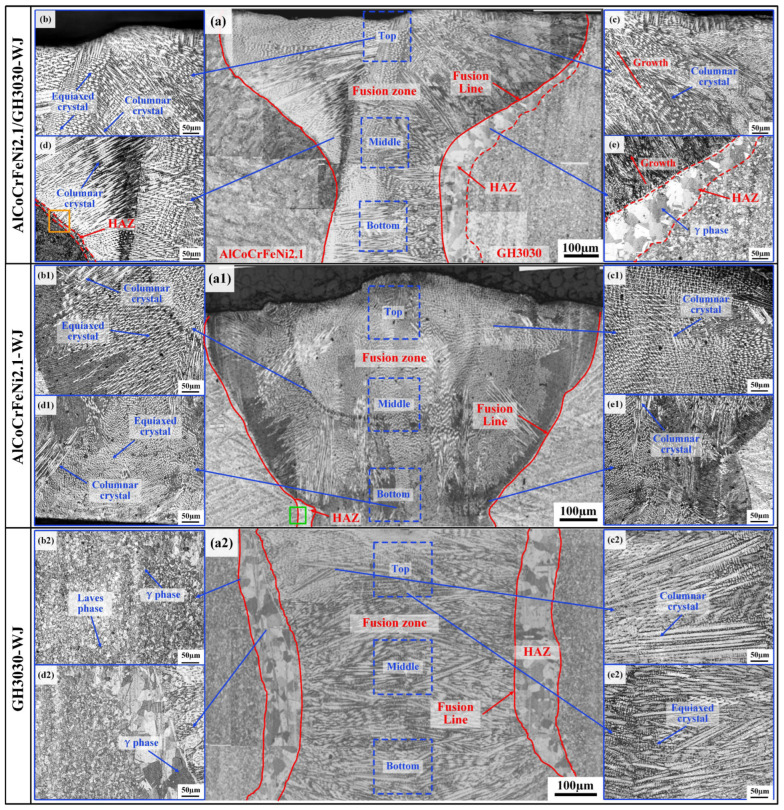
Microstructural morphology of welded joints: (**a**–**e**) Microstructure of the AlCoCrFeNi2.1/GH3030 welded joint (AlCoCrFeNi2.1/GH3030-WJ); (**a1**–**e1**) Microstructure of the AlCoCrFeNi2.1/AlCoCrFeNi2.1 welded joint (AlCoCrFeNi2.1-WJ); (**a2**–**e2**) Microstructure of the GH3030/GH3030 welded joint (GH3030-WJ).

Figure 4 shows the microstructure of the AlCoCrFeNi2.1/GH3030 joint (AlCoCrFeNi2.1/GH3030-WJ), AlCoCrFeNi2.1/AlCoCrFeNi2.1 joint (AlCoCrFeNi2.1-WJ), and GH3030/GH3030 joint (GH3030-WJ) obtained under welding parameters of 1350 W laser power, 1.2 m/min welding speed, and a defocus of 0 mm. Figure 4a–e show the morphology of the AlCoCrFeNi2.1/GH3030 joint. The welded joint exhibits a “nail-like” shape with a uniform and dense microstructure, which is typical of laser welding. The joint consists of three regions: the base material, the heat-affected zone (HAZ), and the fusion zone. The HAZ near the AlCoCrFeNi2.1 side is smaller compared to the GH3030 side, as shown in Figure 4d.

Figure 4b,c are close-up images of the fusion zone. The fusion zone consists of a large number of equiaxed grains and columnar grains, which are randomly distributed. The columnar grains grow most rapidly in the direction of the maximum temperature gradient, i.e., perpendicular to the fusion line. As the cooling rate decreases, the columnar grains in the center of the fusion zone transform into fine equiaxed grains. In laser welding, the microstructure in the HAZ is usually coarser than that of the base material. In this study, the microstructure of the HAZ on the AlCoCrFeNi2.1 side (Figure 4d) does not show significant changes. This is because the welding specimen is relatively thin, with a thickness of only 2 mm, and AlCoCrFeNi2.1 has a low thermal conductivity, making it difficult for laser energy to diffuse into the material. Therefore, there is not enough energy near the HAZ to promote grain growth.

On the GH3030 side (Figure 4e), a distinct heat-affected zone with larger blocky structures is observed. The grain size in the fusion zone is finer compared to the base material and consists of both columnar and equiaxed grains. The grain growth direction near the GH3030 side is relatively uniform, perpendicular to the fusion line. The welded joint interface is well-formed with no porosity or other defects. During solidification, the metal in the molten pool experiences very low undercooling, causing the grains to primarily attach to the semi-molten base material grains and grow towards the center of the welded joint via an epitaxial mechanism. Due to the “preferential growth” of grains, grains oriented along or nearly parallel to the <100> crystallographic direction grow more easily, while grains with other orientations are suppressed. This preferential grain growth results in the structural morphology observed in Figure 4c,e [28].

Figure 4(a1–e1) shows the morphology of the AlCoCrFeNi2.1/AlCoCrFeNi2.1 joint. The joint morphology is similar to the AlCoCrFeNi2.1/GH3030 joint on the AlCoCrFeNi2.1 side, with a smaller heat-affected zone (HAZ). The fusion zone is composed of numerous equiaxed and columnar grains. Figure 4(a2–e2) shows the morphology of the AlCoCrFeNi2.1/AlCoCrFeNi2.1 joint near the GH3030 side, which is similar to the AlCoCrFeNi2.1/GH3030 joint near the GH3030 side. The HAZ is larger, with significantly larger blocky structures, and the fusion zone is also composed of columnar and equiaxed grains.

**Figure 5:**
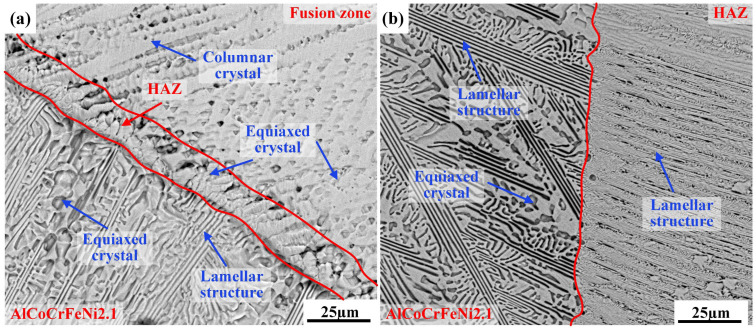
Microstructural morphology: (**a**) Magnified view of the heat-affected zone (HAZ) near the AlCoCrFeNi2.1 side of the AlCoCrFeNi2.1/GH3030 welded joint (orange square in Figure 4d; (**b**) Magnified view of the heat-affected zone (HAZ) of the AlCoCrFeNi2.1/AlCoCrFeNi2.1 welded joint (green square in Figure 4(a1)).

Due to the smaller HAZ on the AlCoCrFeNi2.1 side of the AlCoCrFeNi2.1/GH3030 joint and the AlCoCrFeNi2.1/AlCoCrFeNi2.1 joint, further magnified observations of the HAZs of both joints are shown in Figure 5. From the images, it is evident that the microstructure of the HAZ on the AlCoCrFeNi2.1 side of the AlCoCrFeNi2.1/GH3030 joint differs completely from that of the AlCoCrFeNi2.1/AlCoCrFeNi2.1 joint. Figure 5a shows a magnified view of the HAZ near the AlCoCrFeNi2.1 side of the AlCoCrFeNi2.1/GH3030 joint. The HAZ is mainly composed of equiaxed grains. The part of the fusion zone near the HAZ consists primarily of columnar and equiaxed grains, while the part of the base material near the HAZ is predominantly composed of lamellar structures and equiaxed grains. Figure 5b shows a magnified view of the HAZ of the AlCoCrFeNi2.1/AlCoCrFeNi2.1 joint. Unlike the HAZ of the AlCoCrFeNi2.1/GH3030 joint near the AlCoCrFeNi2.1 side, the HAZ in the AlCoCrFeNi2.1/AlCoCrFeNi2.1 joint is mainly composed of fine lamellar structures, and the base material near the HAZ has a structure similar to that of the AlCoCrFeNi2.1/GH3030 joint near the AlCoCrFeNi2.1 side, mainly consisting of lamellar structures and equiaxed grains.

**Figure 6:**
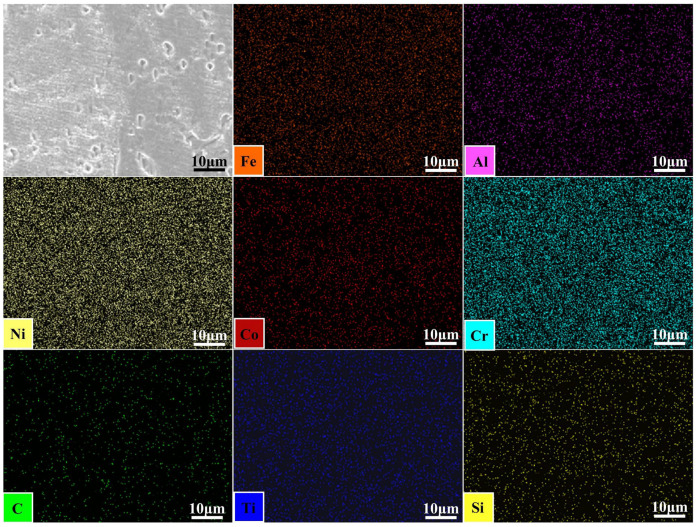
The EDS surface scan image of the weld center for the AlCoCrFeNi2.1/GH3030 welded joint.

**Figure 7:**
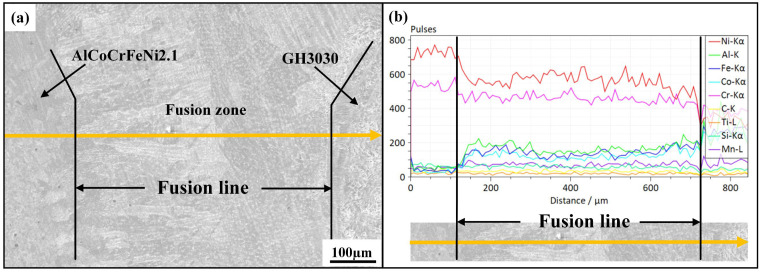
The EDS line scan results for the AlCoCrFeNi2.1/GH3030 welded joint: (**a**) location of the energy spectrum line scan; (**b**) energy spectrum of the line scan.

Figure 6 shows the EDS surface scan of the weld center for the AlCoCrFeNi2.1/GH3030 welded joint. It can be observed that the distribution of elements is relatively uniform, with no evidence of element accumulation at the center or the formation of impurity phases. Figure 7 presents the EDS line scan results for the AlCoCrFeNi2.1/GH3030 joint. The test was conducted along the centerline of the dissimilar joint, from the left side of the GH3030 nickel-based high-temperature alloy base material, passing through the heat-affected zone (HAZ), the weld zone, and then the weld zone on the other end, before reaching the right side of the AlCoCrFeNi2.1 eutectic high-entropy alloy base material. The results show a significant change in element concentrations starting from the fusion line on the GH3030 nickel-based high-temperature alloy side. As the Ni and Cr elements dominate in GH3030, these elements diffuse into the weld zone, resulting in a higher concentration of Ni and Cr in the weld compared to the heat-affected zone of the left-side GH3030 base material. Except for Al and Fe, other trace elements in the GH3030 alloy are uniformly distributed in the weld zone, without any segregation, and the overall concentration is similar to that of the base material, GH3030. In comparison to the AlCoCrFeNi2.1 eutectic high-entropy alloy side, a distinct boundary is observed at the fusion line for the five elements (Al, Co, Cr, Fe, Ni). Due to the higher Ni and Cr content in the GH3030 alloy, these two elements diffuse into the weld zone, where the concentrations of Ni and Cr differ significantly from those in the parent AlCoCrFeNi2.1 eutectic high-entropy alloy. As a result, the concentrations of Al, Co, and Fe in the weld zone of the AlCoCrFeNi2.1 alloy are relatively low.

**Figure 8:**
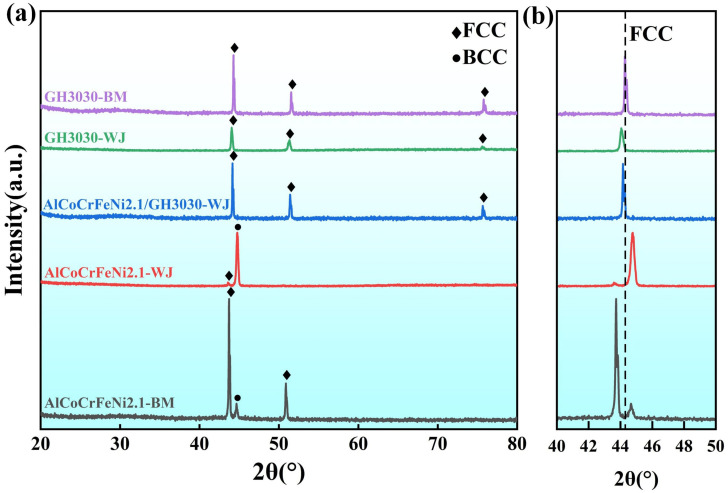
XRD phase analysis spectra: (**a**) XRD spectra for AlCoCrFeNi2.1 base material (AlCoCrFeNi2.1-BM), GH3030 base material (GH3030-BM), GH3030 welded joint (GH3030-WJ), AlCoCrFeNi2.1/GH3030 welded joint (AlCoCrFeNi2.1/GH3030-WJ), and AlCoCrFeNi2.1 welded joint (AlCoCrFeNi2.1-WJ); (**b**) XRD spectra for GH3030 base material (GH3030-BM), GH3030 welded joint (GH3030-WJ), and AlCoCrFeNi2.1/GH3030 welded joint (AlCoCrFeNi2.1/GH3030-WJ) with scanning angle from 30° to 40°.

To further analyze the phase composition of the weld, XRD phase analysis was performed on the AlCoCrFeNi2.1 base material (AlCoCrFeNi2.1-BM), GH3030 base material (GH3030-BM), GH3030 welded joint (GH3030-WJ), AlCoCrFeNi2.1/GH3030 welded joint (AlCoCrFeNi2.1/GH3030-WJ), and AlCoCrFeNi2.1 welded joint (AlCoCrFeNi2.1-WJ), as shown in Figure 8a. The XRD results show that only the FCC structure exists in both the GH3030 base material and the weld zone of the GH3030 welded joint. The primary phase of the AlCoCrFeNi2.1/GH3030 welded joint zone is also FCC. Notably, the main diffraction peak of the FCC phase in the weld zone of both the GH3030 welded joint and the AlCoCrFeNi2.1/GH3030 welded joint shifts to the left compared to the GH3030 base material, as shown in Figure 8b. According to Bragg’s Equation:

2dsinθ = nλ,

where d is the interplanar distance, θ is the diffraction angle, and λ is the wavelength. A decrease in θ indicates an increase in the lattice constant. Studies have shown that with an increase in thermal input, due to thermal expansion and the residual stresses after solidification, the lattice constant increases [29,30]. Therefore, because the thermal input in the weld zones of the GH3030 welded joint and the AlCoCrFeNi2.1/GH3030 welded joint is much higher than in the GH3030 base material, the lattice constant is larger, causing the main diffraction peak of the FCC phase to shift to the left. In addition, during the welding process, Ni and Cr elements from the GH3030 nickel-based superalloy diffused into the weld, while Al, Co, and Fe elements from the AlCoCrFeNi2.1 eutectic high-entropy alloy also migrated toward the weld region. The interdiffusion of these elements within the weld resulted in atomic interactions and lattice occupation, leading to solid-solution strengthening. The severe lattice distortion caused by this process altered the lattice parameters and induced a noticeable shift in the main diffraction peaks.

Furthermore, related research indicates that with the increase in aluminum content, the FCC phase in the alloy tends to transform into the BCC phase [31]. Based on the line scan results (Figure 7), it can be observed that the aluminum content in the AlCoCrFeNi2.1/GH3030 welded joint is relatively low, while the nickel and chromium content are higher. This leads to a distinct phase composition in the AlCoCrFeNi2.1/GH3030 weld, which consists of a single-phase FCC structure, unlike the FCC + BCC mixed-phase structure in the AlCoCrFeNi2.1 base material and AlCoCrFeNi2.1 welded joint.

### 3.3. Microhardness Analysis of AlCoCrFeNi2.1/GH3030 Welded Joint

**Figure 9:**
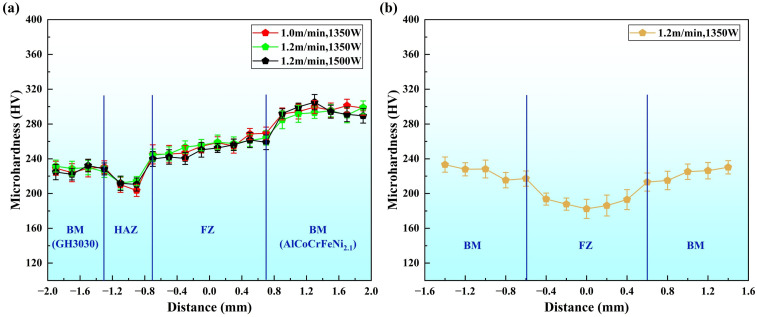
Microhardness of the welded joint under different laser welding process parameters: (**a**) Hardness distribution of AlCoCrFeNi2.1/GH3030 dissimilar material welded joint; (**b**) Hardness distribution of GH3030/GH3030 welded joint.

Figure 9a presents the microhardness test results for the AlCoCrFeNi2.1/GH3030 welded joint under different welding parameters as listed in Table 3. The test was conducted from the GH3030 nickel-based high-temperature alloy side, through the heat-affected zone (HAZ), into the fusion zone (FZ), and then from the other side of the weld into the AlCoCrFeNi2.1 eutectic high-entropy alloy base material (BM). From the figure, it can be observed that the hardness pattern for the AlCoCrFeNi2.1/GH3030 welded joint zone is consistent across the three welding parameters. The hardness value in the heat-affected zone, near the GH3030 nickel-based high-temperature alloy base material side, is the lowest. Under a welding power of 1350 W and a welding speed of 1.0 m/min, the lowest hardness value in the heat-affected zone is 203.6 ± 3.43 HV, due to the highest thermal input causing larger austenitic grain growth. The average hardness in the heat-affected zone is 210.48 ± 4.42 HV. In the weld zone, the hardness increases gradually from the GH3030 side towards the AlCoCrFeNi2.1 eutectic high-entropy alloy side, with only slight fluctuations. The average hardness of the weld is 253.56 ± 2.53 HV. As shown in the XRD spectra (Figure 8), the weld zone is of a face-centered cubic (FCC) structure. As aluminum diffuses from the AlCoCrFeNi2.1 eutectic high-entropy alloy side to the GH3030 nickel-based high-temperature alloy side, the aluminum content is higher near the AlCoCrFeNi2.1 side, leading to an increase in hardness. When comparing the microhardness of the welded joint of the GH3030 nickel-based high-temperature alloy to that of the dissimilar joint, it is evident that no element segregation or impurity phases appear in the dissimilar joint weld, and the element distribution is relatively uniform. Therefore, the performance of the dissimilar joint weld is superior to that of the GH3030 nickel-based high-temperature alloy joint weld, with a higher hardness value than that of the GH3030 nickel-based high-temperature alloy joint (Figure 9b).

### 3.4. Analysis of Welded Joint Performance Based on Indentation Method

#### 3.4.1. Calculation Method for Stress–Strain Curve of Welded Joint Using Nanoindentation

The mechanical properties of the welded joint are critical indicators of whether the joint quality meets the required standards. In this experimental study, due to the small size of the welded specimens, mechanical performance parameters such as tensile, bending, and impact properties could not be obtained. Therefore, nanoindentation testing was used on high-entropy alloys, nickel-based alloys, and dissimilar welded joints to directly obtain performance parameters such as hardness, elastic modulus, plastic work, and elastic work. By analyzing the load–displacement curve data, the stress–strain relationship in the weld zone was determined, and the yield stress of the high-entropy alloy, nickel-based alloy, and dissimilar welded joint was derived through inverse analysis.

**Figure 10:**
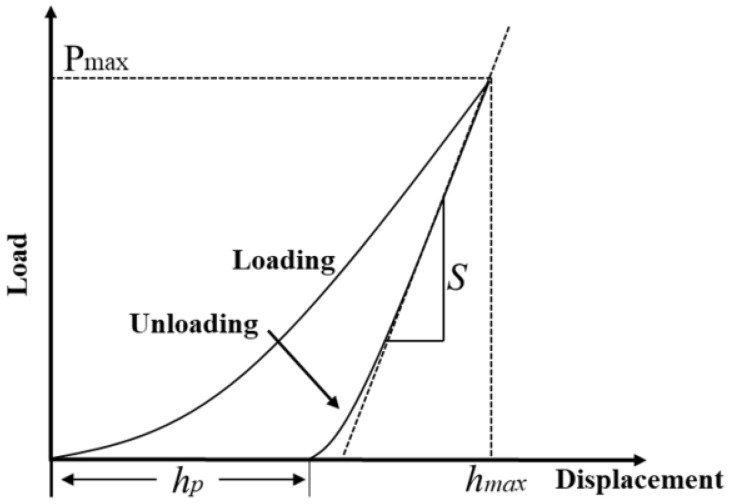
Typical load–displacement curve.

Nanoindentation testing consists of two parts: the loading process and the unloading process. Figure 10 illustrates the complete process of nanoindentation with the classical load–displacement curve. According to the Kick model [32], the relationship between load and displacement can be expressed as follows:

(1) $$P=Ch^{2}$$

where P is the load, h is the loading displacement, and C is the curvature of the loading curve in the loading stage, determined by fitting the loading portion of the curve. In the figure, P_max_ represents the maximum indentation load, *h_max_* denotes the maximum depth of the indentation, and hp represents the permanent plastic deformation after the unloading force. S is the contact stiffness during the unloading phase, defined as the slope of the unloading curve at the beginning of the unloading process.

The power hardening model [33] can describe the elastoplastic behavior of most metallic materials:

(2) $$\left\{\begin{array}{c} \sigma =E\varepsilon ,\sigma \leq \sigma_{y}\\ \sigma =\sigma_{y}{\left(1+\frac{E}{\sigma_{y}}\varepsilon_{p}\right)}^{n},\sigma >\sigma_{y} \end{array}\right.$$

In the expression, E, σ_y_, and n represent the elastic modulus, yield strength, and work hardening index, respectively; ε_p_ is the nonlinear portion of the total strain ε.

When calculating the stress–strain curve using this formula, the elastic modulus, yield strength, and work-hardening index must be determined. The elastic modulus can be directly obtained from the test results, and the remaining parameters can be calculated from the nanoindentation test results.

(1) Determination of Yield Strength σ_y_

Nix and Gao [34] proposed a relationship between hardness and indentation depth based on the Mises yield criterion, which describes the behavior of isotropic metals. The Nix–Gao relation is as follows:

(3) $$\frac{H}{H_{0}}=\sqrt{1+\frac{h^{*}}{h}}$$

In the expression, H is the hardness value measured at an indentation depth of h, h* is a characteristic parameter related to the indenter shape and material properties, and H_0_ is the true hardness of the material, obtained when 1/h approaches 0.

In studies on the relationship between tensile properties and nanoindentation indenter size, Rodríguez et al. [35] proposed the following relationship between hardness and yield strength at the indentation position when using a Berkovich indenter:

(4) $$H_{0}=4.1\sigma_{y}$$

Thus, the yield strength can be determined.

(2) Calculation of the Work Hardening Index n

Dao et al. [36] developed forward and reverse models to calculate material properties. Through simulations of the elastic modulus, strain index, and yield strength for various microstructures, they derived a reverse calculation method for the characteristic stress σ_0.033_ at a nonlinear strain of 0.033. The calculation formula is as follows:

(5) $$\frac{c}{\sigma_{0.03}}=-1.131{\left[\ln(\frac{E_{r}}{\sigma_{0.033}})\right]}^{3}+13.635{\left[\ln(\frac{E_{r}}{\sigma_{0.033}})\right]}^{2}-30.594\left[\ln(\frac{E_{r}}{\sigma_{0.033}})\right]+29.467$$

E_r_ refers to the reduced elastic modulus, which can be directly obtained from the experimental results.

The work hardening index n can be calculated using the following equation:

(6) $$\sigma_{0.033}=\sigma_{y}{\left(1+\frac{E}{\sigma_{y}}0.033\right)}^{n}$$

In their study, Le et al. [37] discovered that the shape factor of the indentation plays a crucial role in determining the calculation results in the above formula. Given the monotonicity of the function, the reverse calculation process is unique; however, errors may arise when fitting the constant C, leading to non-uniqueness in the reverse calculation. To address this, Le et al. [37] optimized the reverse calculation process using the plastic zone r_p_ approach, resulting in a material property equation that is independent of the indenter shape factor and allows for the calculation of the work hardening index. The equation is as follows:

(7) $$\frac{h_{c}}{h_{\max}=0.7+0.55{e^{-2.16n}}^{-40.32\left(\frac{\sigma_{y}}{E}\right)}}$$

In this study, the formula was used to solve for the strain hardening index.

#### 3.4.2. Stress–Strain Curve Calculation and Analysis of the Welded Joint

Nanoindentation tests were performed on the AlCoCrFeNi2.1 eutectic high-entropy alloy base material, GH3030 nickel-based high-temperature alloy base material, AlCoCrFeNi2.1 eutectic high-entropy alloy joint, GH3030 nickel-based high-temperature alloy joint, and AlCoCrFeNi2.1/GH3030 joint. The test sample numbering and corresponding welding parameters are shown in Table 4.

**Figure 11:**
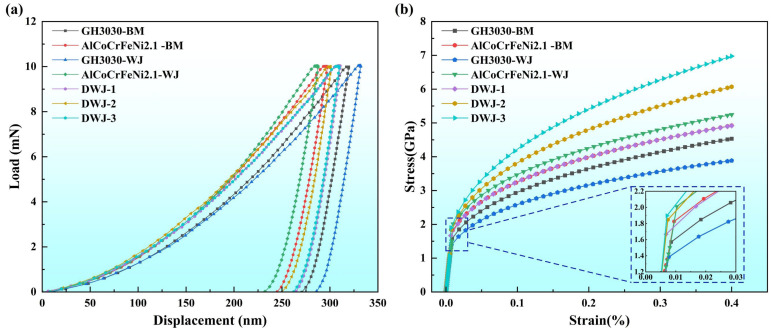
(**a**) Load–displacement curves for GH3030 base material (GH3030-BM), AlCoCrFeNi2.1 base material (AlCoCrFeNi2.1-BM), GH3030 welded joint (GH3030-WJ), AlCoCrFeNi2.1 welded joint (AlCoCrFeNi2.1-WJ), AlCoCrFeNi2.1/GH3030 dissimilar joint weld 1 (DWJ-1) (1350 W, 1.2 m/min), AlCoCrFeNi2.1/GH3030 dissimilar joint weld 2 (DWJ-2) (1500 W, 1.2 m/min), AlCoCrFeNi2.1/GH3030 dissimilar joint weld 3 (DWJ-3) (1350 W, 1.0 m/min). (**b**) Stress–strain curves for GH3030 base material (GH3030-BM), AlCoCrFeNi2.1 base material (AlCoCrFeNi2.1-BM), GH3030 welded joint (GH3030-WJ), AlCoCrFeNi2.1 welded joint (AlCoCrFeNi2.1-WJ), AlCoCrFeNi2.1/GH3030 dissimilar joint weld 1 (DWJ-1) (1350 W, 1.2 m/min), AlCoCrFeNi2.1/GH3030 dissimilar joint weld 2 (DWJ-2) (1500 W, 1.2 m/min), and AlCoCrFeNi2.1/GH3030 dissimilar joint weld 3 (DWJ-3) (1350 W, 1.0 m/min).

Figure 11a shows the nanoindentation load–displacement curves obtained from testing the weld zone based on the test points in Table 4. As can be seen, under different laser welding parameters and at different testing locations, all the curves exhibit similar characteristics. During the stress loading phase, all the curves are nonlinear and follow a quadratic function, indicating that the primary deformation occurring during the test is plastic, with only a small amount of elastic deformation. It is noteworthy that when the welding power is 1350 W and the welding speed is 1.2 m/min, the maximum load–displacement (*h_max_*) for the AlCoCrFeNi2.1 eutectic high-entropy alloy joint is the smallest, indicating that under this welding condition, the material has a stronger resistance to deformation, and its hardness is the highest. In contrast, under the same welding parameters, the GH3030 joint has the largest maximum load–displacement (*h_max_*), indicating weaker resistance to deformation and the lowest hardness.

Based on the load–displacement curves and the relevant parameters from the equipment tests, the data were substituted into Equations (1)–(7) to calculate the stress–strain curve data. The basic mechanical parameters of the weld zone under each welding parameter are calculated and presented in Table 5.

The results show that the elastic modulus of the AlCoCrFeNi2.1 eutectic high-entropy alloy base material, GH3030 nickel-based superalloy base material, AlCoCrFeNi2.1 eutectic high-entropy alloy joint, GH3030 nickel-based superalloy joint, and AlCoCrFeNi2.1/GH3030 joint ranges from 180 to 260 GPa, with the work hardening exponent generally greater than 0.3. During the elastic stage, the mechanical behavior is similar, with GH3030 nickel-based superalloy joints exhibiting relatively low yield strengths, the lowest being 1.07 GPa. The performance of the AlCoCrFeNi2.1/GH3030 joint is generally better than that of the GH3030 nickel-based superalloy joint, with an average yield strength of 1.31 GPa.

Organizational analysis reveals that when laser welding the same material of GH3030 nickel-based superalloy, the performance of the joint decreases due to the presence of impurity phases and elemental segregation in the weld zone. However, after dissimilar welding with a high-entropy alloy, the aggregation of impurity phases and elements in the weld zone is improved, resulting in a performance enhancement. Compared to the AlCoCrFeNi2.1 eutectic high-entropy alloy base material and AlCoCrFeNi2.1 eutectic high-entropy alloy welded joints, the performance of the AlCoCrFeNi2.1/GH3030 welded joint shows a noticeable decrease, likely due to the mismatch in the transformation of dissimilar structures. However, relative to the GH3030 nickel-based superalloy base material and GH3030 alloy joints, the AlCoCrFeNi2.1/GH3030 welded joint still maintains relatively high strength.

By incorporating the data from the table into Equation (2), the stress–strain curve expressions for different parameters were obtained, allowing the stress–strain curves of the weld zone to be derived, as shown in Figure 11b.

From the calculated results (Table 5), the performance advantage of the AlCoCrFeNi2.1/GH3030 joint is primarily reflected in the plastic phase. Due to the high work hardening exponent, the AlCoCrFeNi2.1/GH3030 joint retains a certain level of plasticity after yielding, making it less prone to brittle fracture.

## 4. Conclusions

In this study, dissimilar welding between the AlCoCrFeNi2.1 eutectic high-entropy alloy and the GH3030 nickel-based superalloy was carried out using a fiber laser. The microstructural characteristics and mechanical properties of the dissimilar joints were systematically investigated and compared with those of the GH3030/GH3030 and AlCoCrFeNi2.1/AlCoCrFeNi2.1 joints. Based on the results and analyses, the following conclusions can be drawn:

(1) Microstructural analysis of the AlCoCrFeNi2.1/GH3030 joint under welding conditions of 1350 W power and 1.2 m/min welding speed shows that there is no significant heat-affected zone (HAZ) near the AlCoCrFeNi2.1 side. The fusion zone consists of a large number of equiaxed and columnar grains, with a random distribution. On the GH3030 nickel-based superalloy side, a distinct, larger block-like structure in the HAZ is observed. The fusion zone grains are finer compared to the base material and consist of both columnar and equiaxed grains. The grain growth direction near the GH3030 nickel-based superalloy side is more uniform and perpendicular to the fusion line. No elemental segregation or impurity phases are found at the center of the weld, and the element distribution is relatively uniform.
(2) The hardness values of the AlCoCrFeNi2.1/GH3030 joint weld zone under three welding parameters do not show significant differences. The hardness distribution follows a consistent trend, with an average hardness of 210.48 ± 4.42 HV in the heat-affected zone (HAZ). In the weld zone, the hardness increases gradually from the GH3030 nickel-based superalloy side towards the AlCoCrFeNi2.1 eutectic high-entropy alloy side, with only slight fluctuations. The average hardness of the weld zone is 253.56 ± 2.53 HV. The dissimilar joint weld hardness is higher compared to the GH3030 nickel-based superalloy joint.
(3) The AlCoCrFeNi2.1 eutectic high-entropy alloy joint exhibits the smallest maximum load displacement (*h_max_*), indicating a stronger resistance to deformation and the highest yield strength of 1.566 GPa. The GH3030 joint exhibits the largest maximum load displacement (*h_max_*), indicating weaker resistance to deformation and the lowest yield strength of 1.07 GPa. The AlCoCrFeNi2.1/GH3030 joint outperforms both the GH3030 nickel-based superalloy base material and the GH3030 nickel-based superalloy joint in terms of mechanical properties, with a yield strength of 1.31 GPa. The AlCoCrFeNi2.1/GH3030 joint exhibits a maximum work-hardening exponent of 0.371, which is significantly higher than those of the AlCoCrFeNi2.1/AlCoCrFeNi2.1 and GH3030/GH3030 joints. This indicates that the superior performance of the AlCoCrFeNi2.1/GH3030 joint is primarily manifested in the plastic deformation stage, where it retains considerable ductility after yielding and is less prone to brittle fracture.

This study provides a systematic approach and a feasible technical solution for the development of high-performance dissimilar joints, thereby promoting the application of GH3030 nickel-based superalloy and AlCoCrFeNi2.1 eutectic high-entropy alloy in aerospace, automotive manufacturing, and related fields. Future work will focus on evaluating the high-temperature wear and corrosion resistance of the AlCoCrFeNi2.1/GH3030 dissimilar joints to further explore their applicability in high-temperature environments.

## Figures and Tables

**Table 1:** Chemical Composition of AlCoCrFeNi2.1 Eutectic High-Entropy Alloy (wt%).

Material	Al	Co	Cr	Fe	Ni
AlCoCrFeNi2.1	8.67	18.94	16.71	17.95	37.73

**Table 2:** Chemical Composition of GH3030 High-Temperature Nickel-Based Alloy (wt%).

Material	C	Cr	Ni	Ti	Al	Fe	Mn	Si	P	S
GH3030	0.10	21.20	58.11	17.94	0.02	1.20	0.60	0.69	0.02	0.02

**Table 3:** Welding Process Parameters.

Sample	Welding Material	Laser Power (W)	Welding Speed (m/min)	Heat Input (J/mm)	Defocus Amount (mm)	Argon Gas Flow Rate (L/min)
1	GH3030/GH3030	1350	1.2	67.5	0	16
2	AlCoCrFeNi2.1/AlCoCrFeNi2.1	1350	1.2	67.5	0	16
3	AlCoCrFeNi2.1/GH3030	1350	1.2	67.5	0	16
4	AlCoCrFeNi2.1/GH3030	1500	1.2	75	0	16
5	AlCoCrFeNi2.1/GH3030	1350	1.0	81	0	16

**Table 4:** Nanoindentation Test Point Numbering and Corresponding Welding Parameters.

Sample	Experimental Material	Laser Power (W)	Welding Speed (m/min)
1	GH3030 base material	—	—
2	AlCoCrFeNi2.1 base material	—	—
3	GH3030 welded joint	1350	1.2
4	AlCoCrFeNi2.1 welded joint	1350	1.2
5	AlCoCrFeNi2.1/GH3030 welded joint 1	1350	1.2
6	AlCoCrFeNi2.1/GH3030 welded joint 2	1500	1.2
7	AlCoCrFeNi2.1/GH3030 welded joint 3	1350	1.0

**Table 5:** Basic mechanical parameters of various experimental materials.

Sample	Experimental Material	Er (GPa)	H (GPa)	E (GPa)	σy (GPa)	n	εy
1	GH3030 base material	176.66	4.99	184.85	1.20	0.32	0.0065
2	AlCoCrFeNi2.1 base material	174.21	5.87	189.33	1.41	0.31	0.0075
3	GH3030 welded joint	174.82	4.44	186.23	1.07	0.30	0.0058
4	AlCoCrFeNi2.1 welded joint	242.44	6.50	187.00	1.57	0.31	0.0084
5	AlCoCrFeNi2.1/GH3030 welded joint 1	232.58	5.29	266.42	1.28	0.30	0.0048
6	AlCoCrFeNi2.1/GH3030 welded joint 2	231.06	5.69	255.59	1.37	0.34	0.0054
7	AlCoCrFeNi2.1/GH3030 welded joint 3	176.66	5.37	253.91	1.29	0.371	0.0051

## Data Availability

The original contributions presented in this study are included in the article. Further inquiries can be directed to the corresponding authors.
